# Factor Structure and Measurement Invariance for the Cognitive Emotion Regulation Questionnaire (CERQ) Among Women Newly Diagnosed With Breast Cancer

**DOI:** 10.3389/fpsyg.2019.01132

**Published:** 2019-05-22

**Authors:** Lingyan Li, Shichen Li, Yuping Wang, Yanjie Yang, Xiongzhao Zhu

**Affiliations:** ^1^Medical Psychological Center, The Second Xiangya Hospital, Central South University, Changsha, China; ^2^School of Nursing, Nanchang University, Nanchang, China; ^3^School of Humanities and Social Sciences, Xi’an Jiaotong University, Xi’an, China; ^4^School of Public Health, Harbin Medical University, Harbin, China

**Keywords:** cognitive emotion regulation, breast cancer, factor structure, measurement invariance, confirmatory factor analysis

## Abstract

**Background:**

Given that emotion regulation counts for much in breast cancer, it is important to fully understand its construct. The cognitive emotion regulation questionnaire (CERQ) is a widely applied instrument for measuring conscious cognitive coping strategies in both general and clinical samples; however, there are no data on its factor structure in women with breast cancer, not to mention evidence of measurement invariance (MI) across sociodemographic variables. Thus, the purpose of the present study was to examine the latent factor structure and MI between different sociodemographic groups for CERQ in specific patients.

**Methods:**

The sample consisted of 1032 women newly diagnosed with breast cancer, with a mean age of 47.54 years (*SD* = 8.51). The latent factor structure for CERQ was tested using confirmatory factor analysis (CFA). Further, MI various sociodemographic variables was evaluated by a series of multiple-group CFA process.

**Results:**

The nine-factor CFA model was an adequate fit for the data collected in women with breast cancer. Also, this nine-factor structure had strong factorial invariance across age, place of residence, educational levels, and employment status.

**Conclusion:**

This study firstly examined the latent factor structure for CERQ among Chinese women with malignancy and MI across various sociodemographic variables, which deepens the understanding of the construct for CERQ as a useful tool for assessing patients’ conscious cognitive component of emotion regulation based on self-report.

## Introduction

In 2018, it was estimated that there were approximately 2.1 million new cases of female breast cancer worldwide (Bray et al., 2018) – about 11.4% of these cases were in younger women, defined here as those under 45 years of age (Howlader et al., 2015), accounting for the largest proportion of female cancer death in China (Chen et al., 2016). The diagnosis and treatment of breast cancer are continuous traumatic and stressful experiences that can evoke a variety of emotional distress (Conley et al., 2016). The pattern by which women deal with their negative emotions, especially the cognitive aspect of emotion regulation, plays a significant role in the process of patients’ adaption to cancer (Kvillemo and Bränström, 2014; Wang et al., 2014; Li et al., 2015).

In 2001, attempting to fully understand the cognitive element of emotion regulation, Garnefski et al. (2001) developed a new questionnaire by conducting principal component analyses of data collected from a sample of university students, and named it the cognitive emotion regulation questionnaire (CERQ). It consists of 36 items, assessing nine trait-like conscious cognitive coping strategies that an individual adopts when facing negative life events (Garnefski et al., 2001). The first two strategies, Self-blame and Blaming others, reflecting on that individual, have a thought that the primary cause of the negative event should be attributed to oneself or others; Acceptance refers to the cognitive process related to having thoughts of acceptance and resignation of the negative situation; Refocusing on planning refers to concentrating on the measures to adopt in dealing with the unpleasant situation; Positive refocusing can be interpreted as one’s attempt to focus attention on happy thoughts rather than the stressful event; Rumination means overthinking about the consequences of the negative event; Positive reappraisal refers to looking on the bright side of a negative situation; putting into perspective is the strategy that an individual compares the distasteful thing to others or mulls over its influence with time; and finally, catastrophizing is described as anticipatory thoughts that exaggerate the level of negativity of what happened (Garnefski et al., 2001). Besides, a second-order Principal Component Analysis resulted in two higher order factors: one was called “more adaptive strategies,” including five first-order factors: positive refocusing, positive reappraisal, putting into perspective, refocus on planning, and acceptance; the remaining factor was termed “less adaptive strategies,” which included four first-order factors: self-blame, rumination, catastrophizing, and other-blame.

The nine-factor CERQ has been evidenced to have excellent psychometric properties across languages from different cultures, such as European cultures, like French (Jermann et al., 2006), Hungarian (Miklósi et al., 2011), and Spanish (Domínguez-Sánchez et al., 2013); Asian cultures, like Chinese (Zhu et al., 2008); Middle Eastern cultures, like Arabic (Megreya et al., 2016), Turkish (Tuna and Bozo, 2012), and Persian (Abdi et al., 2012); and South America cultures, like Brazilian (Schäfer et al., 2018), Peruvian (Dominguez and Medrano, 2016), and Argentinian (Medrano et al., 2013). More specifically, except for general populations, the CERQ has been applied to multiple clinical populations, such as fibromyalgia syndrome population (Feliu-Soler et al., 2017) and the psychiatric disorder population (Garnefski and Kraaij, 2006), showing adequate reliability and validity. Regarding the second-order factor structure for the CERQ, the results of previous studies are inconsistent. The second-order factor model showed appropriate global fit indices among the general population, both for the French version (Jermann et al., 2006) and the Spanish version (Domínguez-Sánchez et al., 2013). In terms of being applied to the Arabic population (Megreya et al., 2016), as well as in a sample of patients with fibromyalgia (Feliu-Soler et al., 2017), the model fit was acceptable but took a turn for the worse after including the two second-order factors, whereas a second-order model did not fit the data at all among a sample of Brazilian university students (Schäfer et al., 2018). In one study (van Wijk-Herbrink et al., 2011), three higher-order factors of the nine CERQ subscales were extracted instead of two.

In a nutshell, the CERQ is an adaptable instrument for evaluating the emotion regulation in a variety of samples. There was already evidence suggesting that women with breast cancer showed special patterns of using cognitive emotion regulation strategies (Li et al., 2015), and some strategies had an important effect on patients’ emotions and quality of life (Wang et al., 2014). However, to the best of our knowledge, no study has been carried out to examine the factor structure properties of the CERQ among women with breast cancer. Previous practices in the context of an undefined structure for CERQ among these patients may cause biased findings. Taking this into consideration, the primary purpose of the present study was to examine the fitness of the first-order and high-order factor model for CERQ in a sample of Chinese women who had recently been diagnosed with breast cancer. In addition, studies have revealed differences in some sociodemographic variables, such as age, education, and place of residence in reported use of cognitive emotion regulation strategies, both in the general population (Garnefski and Kraaij, 2006), and in a sample of breast cancer patients (Li et al., 2017). However, so far, none of the existing studies have tested the measurement invariance (MI) of CERQ across these variables, which is critical in ensuring the comparability of subscale scores between different sociodemographic groups. Thus, another purpose of this study was to evaluate the equivalence of measurement models across various sociodemographic variables.

## Materials and Methods

### Patients

From March 2011 to March 2016, women who had recently received a diagnosis of breast cancer from two hospitals in Changsha city were asked to take part in the present study. The inclusion criteria were as follows: (1) aged 18 to 70, (2) had an initial diagnosis of breast cancer through biopsy during the last month, and (3) can read Chinese. Patients with a known major medical condition that required treatment (other than breast cancer), a known major psychiatric disorder, or a history of substance abuse, were excluded. In total, 1124 patients were interested in participating; however, five patients declined before giving informed consent, four patients were excluded because of a recurrence of breast cancer, and 83 patients could not complete the questionnaires, with the final sample amounting to 1032 patients.

### Instruments

The 36-item CERQ was used to evaluate cognitive strategies adopted by individuals when they face threatening or stressful situation. It includes nine conceptually different subscales, each consisting of four items: acceptance, positive refocusing, refocusing on planning, positive reappraisal, putting into perspective, self-blame, rumination, catastrophizing and blaming others. Each item response was measured on a five-point Likert scale, with 1 indicating almost never, and 5 meaning almost always. The higher the scores on subscales, the greater the use of a certain cognitive coping strategy. The Chinese version of CERQ (Zhu et al., 2008) was applied in this study, showing good to excellent internal consistency for the subscales.

### Data Collection

The research was conducted in the hospital ward. Participants received a copy of the printed questionnaire immediately after providing informed consent. They were told to fill in the CERQ on the spot on their own and that there was a trained psychology student nearby who could provide professional assistance if they had some questions about the CERQ.

### Statistics

Mplus 7.4 software was used to conduct data analyses (Muthén and Muthén, 2012). While keeping pre-existing findings on the construct of CERQ in mind, the original nine-factor structure model (model 1) was tested through a confirmatory factor analysis (CFA) in the pooled sample of women who were newly diagnosed with breast cancer. In view of the general goodness of fit for model 1 and related modification indices, a modified nine-factor structure model was carried out as model 2. We then conducted a CFA to test the fit of the second-order factor structure model based on model 2. By comparing the indices of first-order and second-order factor structures of CERQ, the best-fitted factor structure was determined in the pooled sample, which was taken as the basic factor structure in multi-group MI analysis.

A series of multi-group CFAs of CERQ were conducted to determine the extent to which the factor structure was comparable across age, places of residence, levels of education, and employment status (i.e., younger and older, urban and rural, different levels of education, employed, and unemployed). According to Muthén and Muthén, 2012, three steps for MI of ordinal variables should be considered, namely, the configural model, the metric model (item factor loading invariance) and the scalar model (item threshold invariance), from least restrictive to most restrictive. However, when a factor indicator has cross-loading on factors, use of the metric model is not allowed.

Given the ordinal metric nature of the items, the polychoric correlations were used to estimate the models instead of the covariance matrix, which may underestimate the true extent of relationships among items. The weighted least squares means and variance adjusted (WLSMV) estimator was chosen for all analyses, which were performed well on categorical data and also in a complicated model and missing data condition. The Mplus DIFFTEST option was used to conduct chi-square (χ^2^) difference tests for the nested model comparison evaluation. Owing to the fact that it is easy to achieve a significant result of χ^2^ tests in large sample sizes (Wen et al., 2004), some fitting indices were used as criteria for a good model fit: the comparative fit index (CFI), the non-normed fit index (NNFI), the root mean square error of approximation (RMSEA), and the standardized root mean squared residual (SRMR). According to Marsh et al. (2014), CFI ≥ 0.90, NNFI ≥ 0.90, RMSEA ≤ 0.08, and SRMR ≤ 0.08 indicate models with an acceptable fit. In terms of model comparison, χ^2^ change and CFI change were reviewed. As the χ^2^ change test is also sensitive in large sample sizes, the CFI change was more important for judging model improvement because it is independent of sample size and not correlated with the overall fit measurements (Vandenberg and Lance, 2000). As suggested by Cheung and Rensvold (2002), a reduction of less than 0.01 in CFI indicates no rejection of the null hypothesis that models are similar.

## Results

### Participants’ Background

The basic information of the 1032 participants in the current study is presented in Table 1. The age of these participants ranged from 25 to 70 years (mean age = 47.54 years and *SD* = 8.51); among these patients were 415 patients aged below 45 years and 617 patients aged 45 and above. Those from urban and rural areas (531 and 501, respectively) each comprised about half the sample size. The vast majority of patients (93.3%) were married, 3.6% were divorced, 2.5% were widowed, and 0.6% were single. On average, they had finished 9.91 years of schooling; 264 patients received basic education, 581 finished high school, and 187 obtained a college degree or above. As regards the employment situation, 75.2% were employed, 14.8% were housewives, and 10% were retired. All patients were under treatment when taking the investigative questionnaire: 14.5% were undergoing chemotherapy before mastectomy, 22.5% of the patients had just received a mastectomy and were undergoing anti-inflammatory therapies, and 63% had been undergoing adjuvant therapy after mastectomy.

**Table 1:** Demographic and medical data of the study sample (*n* = 1032).

	M/*n*	%
Years of age (SD)	47.54 (8.51)	
Years of schooling (SD)	9.91 (3.67)	
Place of residence		
Urban	531	51.5
Rural	501	48.5
Marital status		
Married	963	93.3
Widowed	26	2.5
Divorced	37	3.6
Single	6	0.6
Employment status		
Employed	776	75.2
Housewife	153	14.8
Retired	103	10.0
Stage of disease		
I	146	14.2
II	791	76.6
III	91	8.8
IV	4	0.4
Weeks since diagnosis (SD)	1.17 (0.61)	
Therapy type		
Mastectomy	232	22.5
Chemotherapy	150	14.5
Mastectomy with chemotherapy	594	57.6
Mastectomy with radiotherapy	15	1.4
Mastectomy with endocrinotherapy	41	4.0

### Factor Structure of CERQ

The model fit indices of the factor structure model for the full sample were listed in Table 2. As can be seen, the CFA of the original nine-factor model resulted in the following global fit indices: χ^2^ (df) = 5898.992 (558), *p* < 0.001; RMSEA = 0.096 with an interval at 90% (0.094–0.099); CFI = 0.980; NNFI = 0.978 and SRMR = 0.058. Although the CFI and NNFI were both >0.95 and the SRMR value was <0.08, model l had not yet reached a good fit in respect of the value of RMSEA, which was >0.08. It was found that this poor fit might be due to some item cross-factor loadings through the review of the modification indices. After Item 25 “I think that it all could have been much worse” and Item 27 “I think that it hasn’t been too bad compared to other things” being reassigned both to the dimensions of putting into perspective and Catastrophizing, all of the fit indices of this new model (model 2) were better and at an acceptable level, with χ^2^ (df) = 3312.127 (556), *p* < 0.001; RMSEA = 0.069 with an interval at 90% (0.067–0.072); CFI = 0.990; NNFI = 0.988; and SRMR = 0.039. Also, the significant change of χ^2^ of 2586.865 for 2 degrees of freedom indicates that model 2 is a significant improvement on model 1.

**Table 2:** Model fitting indices for first-order and second-order factor modeling in the pooled sample, *n* = 1032.

Model	χ^2^ (df), *p* =	Δχ^2^ (df), *p* =	RMSEA (90% CI)	CFI	NNFI	SRMR
Model 1	5898.992 (558), *p* < 0.001		0.096 (0.094–0.099)	0.980	0.978	0.058
Model 2	3312.127 (556), *p* < 0.001	2586.865 (2), *p* < 0.001^a^	0.069 (0.067–0.072)	0.990	0.988	0.039
Model 3	4676.916 (582), *p* < 0.001	1364.789 (26), *p* < 0.001^b^	0.083 (0.080–0.085)	0.985	0.983	0.083

*Mode 1, the original nine factor first-order model; Model 2, the modified nine factor model in which Item 25 and Item 27 have cross-loading on the dimensions of putting into perspective and catastrophizing; Model 3, the second-order model based on model 2; a, model 2 vs. model 1; b, model 3 vs. model 2; RMSEA, root mean square error of approximation; CFI, comparative fit index; NNFI, non-normal fit index; SRMR, standardized root mean squared residual.*

The second-order model (model 3) CFA was subsequently carried out on the basis of model 2. After the inclusion of two high-order factors, the CFA model resulted in a worse fit to the data, with all the indices being worse than that in the first-order CFA model, especially the RMSEA and SRMR, which were both >0.08. These results indicated that the best model for the full sample was the modified nine-factor model. The completely standardized item loading for the structure of models 1 and 2 are presented in Table 3.

**Table 3:** Item factor loading for CERQ factors (model 2), *n* = 1032.

Item	Factor 1	Factor 2	Factor 3	Factor 4	Factor 5	Factor 6	Factor 7	Factor 8	Factor 9
Item 1	**0.939 (0.939)**								
Item 2	**0.663 (0.663)**								
Item 3	**0.975 (0.975)**								
Item 4	**0.966 (0.966)**								
Item 5		**0.965 (0.965)**							
Item 6		**0.983 (0.982)**							
Item 7		**0.541 (0.541)**							
Item 8		**0.936 (0.936)**							
Item 9			**0.869 (0.869)**						
Item 10			**0.940 (0.939)**						
Item 11			**0.971 (0.971)**						
Item 12			**0.971 (0.971)**						
Item 13				**0.916 (0.916)**					
Item 14				**0.974 (0.973)**					
Item 15				**0.967 (0.967)**					
Item 16				**0.947 (0.947)**					
Item 17					**0.948 (0.948)**				
Item 18					**0.977 (0.977)**				
Item 19					**0.963 (0.963)**				
Item 20					**0.945 (0.945)**				
Item 21						**0.951 (0.950)**			
Item 22						**0.940 (0.940)**			
Item 23						**0.914 (0.914)**			
Item 24						**0.932 (0.932)**			
Item 25							**0.977 (0.250)**	**(0.609)**	
Item 26							**0.697 (0.780)**		
Item 27							**0.466 (1.240)**	**(−0.677)**	
Item 28							**0.801 (0.871)**		
Item 29								**0.936 (0.935)**	
Item 30								**0.949 (0.949)**	
Item 31								**0.935 (0.935)**	
Item 32								**0.960 (0.960)**	
Item 33									**0.915 (0.915)**
Item 34									**0.921 (0.921)**
Item 35									**0.976 (0.976)**
Item 36									**0.982 (0.982)**

*Factor 1-factor 9 were self-blame, acceptance, rumination, positive refocusing, refocusing on planning, positive reappraisal, putting into perspective, catastrophizing, and blaming others, respectively; all loadings are statistically significant at p = <0.001 level; all loadings in bold are the loadings of items that belongs to the specific factor.*

Before the MI analysis, the modified nine-factor model was tested on different age, place of residence, education level, and employment status groups. Indices revealed that the modified nine-factor model generally fit the data well in each subsample. Thus, the modified nine-factor model can serve as the initial model for the subsequent MI tests.

### Measurement Invariance Across Sociodemographic Groups

Table 4 contains the fit indices for the basic model in subsamples and each MI test step. Since there were two indicators loading on two factors, only the configural invariance model and scalar invariance model were tested on each sociodemographic variable. To determine whether patient age affected the measurement model, the patients were split into younger and older groups based on the cutoff age of 45, according to the evidence that women aged below 45 are believed to suffer from cancer at a “young age” (Howlader et al., 2015). Results of the configural invariance model indicated that the nine-factor structure with similar correspondence between items and factors was verified across age groups [χ^2^ (df) = 3769.327 (1112), *p* < 0.001; RMSEA = 0.068 with an interval at 90% (0.066–0.070); CFI = 0.990; NNFI = 0.989; SRMR = 0.045]. For threshold invariance across age groups, the model fitted the data well [χ^2^ (df) = 3765.502 (1240), *p* < 0.001; RMSEA = 0.063 with an interval at 90% (0.061–0.065); CFI = 0.991; NNFI = 0.991]. Though the DIFFTEST showed a significant χ^2^ (df) change, the 0.001 CFI increase from the invariance configural model showed that the constrained model was not rejected.

**Table 4:** Measurement invariance model fitting indices and comparison.

Invariance model	χ^2^ (df), *p* =	RMSEA (90% CI)	CFI	NNFI	SRMR	Model comparison	Δχ^2^ (df), *p* = ^c^	ΔCFI
Age								
Younger (*n* = 415)	1695.860 (556), *p* < 0.001	0.070 (0.066–0.074)	0.987	0.986	0.049			
Older (*n* = 617)	2131.427 (556), *p* < 0.001	0.068 (0.065–0.071)	0.992	0.991	0.041			
1. Configural	3769.327 (1112), *p* < 0.001	0.068 (0.066–0.070)	0.990	0.989	0.045			
2. Threshold	3765.502 (1240), *p* < 0.001	0.063 (0.061–0.065)	0.991	0.991	0.045	2 vs. 1	244.049 (128), *p* < 0.001	0.001
Place of residence								
Urban (*n* = 531)	1893.713 (556), *p* < 0.001	0.067 (0.064–0.071)	0.990	0.988	0.045			
Rural (*n* = 501)	2037.855 (556), *p* < 0.001	0.073 (0.070–0.076)	0.989	0.987	0.044			
1. Configural	3907.756 (1112), *p* < 0.001	0.070 (0.067–0.072)	0.988	0.987	0.045			
2. Threshold	3954.577 (1240), *p* < 0.001	0.065 (0.063–0.067)	0.989	0.988	0.046	2 vs. 1	304.406 (128), *p* < 0.001	0.001
Level of education								
Basic (*n* = 264)	1229.631 (556), *p* < 0.001	0.068 (0.063–0.073)	0.993	0.992	0.048			
Secondary (*n* = 581)	2079.327 (556), *p* < 0.001	0.069 (0.066–0.072)	0.990	0.989	0.042			
High (*n* = 187)	1159.141 (556), *p* < 0.001	0.076 (0.070–0.082)	0.985	0.983	0.063			
1. Configural	4248.124 (1668), *p* < 0.001	0.067 (0.065–0.070)	0.991	0.990	0.048			
2. Threshold	4443.057 (1924), *p* < 0.001	0.062 (0.059–0.064)	0.992	0.992	0.049	2 vs. 1	502.380 (256), *p* < 0.001	0.001
Employment status								
Employed (*n* = 776)	2760.916 (556), *p* < 0.001	0.071 (0.069–0.074)	0.990	0.988	0.043			
Unemployed (*n* = 256)	1266.482 (556), *p* < 0.001	0.071 (0.066–0.076)	0.987	0.985	0.052			
1. Configural	3721.588 (1112), *p* < 0.001	0.067 (0.065–0.070)	0.990	0.989	0.045			
2. Threshold	3688.185 (1240), *p* < 0.001	0.062 (0.060–0.064)	0.991	0.990	0.046	2 vs. 1	288.116 (128), *p* < 0.001	0.001

*RMSEA, root mean square error of approximation; CFI, comparative fit index; NNFI = non-normal fit index; Δχ^2^ = change in chi-square; c = Δχ^2^ test is conducted using the DIFFTEST option for the nested models; ΔCFI, change in comparative fit index.*

To evaluate MI across living areas, women were classed into an urban group and a rural group by their usual place of residence. The results of the configural invariance model and the threshold invariance model showed a general good fitness, with χ^2^ (df) = 3907.756 (1112) and 3954.577 (1240), respectively, while the RMSEA had an interval of 90% = 0.070 (0.067–0.072) and 0.065 (0.063–0.067), respectively, with both CFI > 0.95 (0.988 vs. 0.989) and both NNFI > 0.95 (0.987 vs. 0.988). An increase in the CFI value of 0.001 showed that the invariance threshold model did not worsen the unconstrained model.

In terms of testing invariance across education levels, women were grouped into three subsamples, i.e., basic education, secondary education, and high education. The invariance configural model showed an adequate fit, RMSEA had an interval of 90% = 0.067 (0.065–0.070), with both CFI and NNFI < 0.95 and SRMR < 0.05. The invariance threshold model showed similar fit indices, and this constrained model could be assumed by the evidence of the 0.001 CFI increase compared to the configural model.

In order to examine the invariance between different employment statuses, women who had a job at the time of the survey were classified under the employed group, whereas housewives, and retired women were classified under the unemployed group. The fit indices supported the invariance configural model, as did the RMSEA with an interval of 90% = 0.067 (0.065–0.070), CFI = 0.990, NNFI = 0.989, and SRMR = 0.045. With the evidence of the 0.001 CFI increase, it could be assumed that the threshold was an invariance across employment statuses.

## Discussion

The CERQ is a widely used instrument for measuring the conscious use of cognitive emotion regulation strategies in general and clinical populations. Though the factor structure of CERQ has been examined in various samples, it remains unknown in women with breast cancer, who showed a specific style of using the cognitive coping strategies. Moreover, there are no professional tests of sociodemographic-crossed MI for CERQ, such as age, place of residence, education levels, and employment status. Therefore, the factor structure of the CERQ and the MI between various groups in Chinese women with breast cancer was examined in this study. It was found that the original nine-factor structure with item 25 and item 27 cross-factor loading in putting into perspective and Catastrophizing fitted the data well. The result indicated that in the context of newly diagnosed cancer, where patients usually focus their attention on the bad news of the disease, the items “I think that it all could have been much worse” and “I think that it hasn’t been too bad compared to other things” would also measure catastrophizing. This result, to some degree, may partly explain the finding that women with breast cancer who reported more frequent use of the putting into perspective had a worse perceived quality of life in the previous study (Li et al., 2015).

Fitting indices comparison between the first-order factor model and the second-order factor model reveals that the nine conceptual strategies could not be simply summarized into adaptive and less adaptive strategies suggested by an earlier study (Garnefski et al., 2001). Similar to findings in later studies (Garnefski and Kraaij, 2007), the dichotomic classification of defining the functionality of cognitive strategies may not be suitable when applied to a life-threatening situation. Future studies should carefully classify which coping strategies used by women with breast cancer are beneficial or harmful.

Measurement invariance across a sociodemographic sample is fundamental in making a reliable comparison of the CERQ scores between patients with different sociodemographic characteristics in order to obtain a valid statistical inference. The configural invariance indicates that the conceptual framework to define the nine latent factors formed by the 36 items of CERQ is the same for patients of different ages, residence areas, levels of education, and employment status. The item threshold invariance, along with the configural invariance model, and demonstrates the presence of strong factorial invariance for CERQ on various sociodemographic variables. In other words, it makes sense to operate the mean score comparison between patients with younger and older age, patients from urban and rural areas, patients with different levels of education, and patients in different employment conditions (Cheung and Rensvold, 2002).

The present study should be interpreted with several limitations. Firstly, participants in our study were all women who were newly diagnosed and undergoing treatment for breast cancer; therefore, the results apply only to patients who were coping with breast cancer in the short term. Secondly, due to the nature of the cross-sectional design, the results of our study cannot certify whether participants responded to CERQ stably across time. Future research with prospective investigation should be conducted to answer this question.

In short, despite the above-mentioned limitations, our findings helped deepen our understanding of the factor structure of cognitive emotion regulation among Chinese women with breast cancer. To the best of our knowledge, our study was the first to attempt an evaluation of MI across a series of sociodemographic variables. It is confirmed that this nine latent factor structure for CERQ has sociodemographic-crossed strong factorial MI.

## Ethics Statement

This study was approved by the Ethics Committee of the Second Xiangya Hospital, Central South University, Changsha, China. All subjects gave written informed consent in accordance with the Declaration of Helsinki.

## Author Contributions

LL, YY, and XZ conceived and designed the study. YW organized and supervised the data collection. LL and SL collected and inputted the data. LL carried out the data analysis and drafted the manuscript. SL and YW provided the critical comments on various drafts of the manuscript. All authors read and approved the final manuscript.

## Conflict of Interest Statement

The authors declare that the research was conducted in the absence of any commercial or financial relationships that could be construed as a potential conflict of interest.
